# Prostate-MRI reporting should be done with the aid of AI systems: Pros

**DOI:** 10.1007/s00330-024-10909-y

**Published:** 2024-07-09

**Authors:** Tobias Penzkofer

**Affiliations:** 1 Department of Radiology, Charité Universitätsmedizin Berlin, Berlin, Germany; 2 Berlin Institute of Health, Berlin, Germany

## Introduction

Prostate magnetic resonance imaging (MRI) has become an integral part of how prostate cancer is diagnosed and monitored. The prostate MRI pathway has been shown to reduce the number of biopsies and the detection of Gleason grade group ≤ 2 cancers, to the benefit of patients. However, if we want to expand on this premise and even venture into using prostate MRI for screening, new challenges will soon arise. Radiologists and technicians are already scarce and, if the number of prostate MRIs increases, even with shorter examination times (thanks to deep learning reconstruction), handling this workload volume becomes a critical issue. This opinion piece makes the case for artificial intelligence (AI) in prostate MRI reporting as a possibility, opportunity, and even necessity to tackle this problem.

Please note that some of the presented arguments are purposedly one-sided. This commentary should be read in conjunction with the companion commentary [1] making the case against using AI in prostate MRI reporting.

## The case for AI in prostate-MRI reporting

First, AI brings several convenience factors to prostate MRI reporting. Automatic prostate segmentation and volumetry are features with which almost every artificial intelligence product for prostate MRI is equipped, allowing for automatic calculation of PSA (prostate specific antigen) density, potentially with higher fidelity compared to the traditional ellipsoid formula [2]. Additionally, automatic structured report creation from AI annotations and segmentation, even if revised by the reader, can contribute to the clarity and consistency of reports [3].

We know that prostate MRI reporting comes with a substantial degree of interreader variability, including easy tasks like prostate MRI segmentation, but also for PI-RADS (Prostate Imaging Reporting and Data System) assessment [4]. This variability is a function of readers’ experience, with less experienced readers showing more pronounced interreader variability compared to more experienced ones [5]. As the AI algorithms used in prostate MRI applications are deterministic by nature, significant  variability exists only between different systems. A given application will always perform at the same level and is never tired or distracted. It has been shown that especially less experienced readers, can benefit from these systems [6, 7], potentially lowering the error rate and freeing up resources for supervising expert readers. True, this evidence is mostly retrospective in nature, but studies like the PI-CAI (Prostate Imaging -Cancer AI) challenge have impressively shown the potential of AI with a large number of cases and readers [8]. Surely prospective, randomized controlled trials should be performed as well, but as with many technological developments, this can be done more easily once the adoption rate of the technology is high.

The ongoing discussion about the use of Gadolinium-based dynamic contrast-enhanced (DCE) imaging is another potential benefit of using AI for prostate MRI. The available systems have almost exclusively been trained on biparametric MRI. Therefore, the broad application of AI in prostate MRI could open the door to finally abandoning Gadolinium-based imaging. This change would reduce costs as well as the harm to patients and the environment. This could be especially useful in serial examinations when used for monitoring prostate cancer patients on active surveillance [9], and will surely be necessary in a screening setting, in case this will be instituted, as scanner time will be at a premium if the entire at-risk population is to be examined.

Another advantage of an AI-supported prostate reading system is the potential to support the entire prostate diagnosis workflow from acquisition, reading, and reporting, up to the planning of the biopsy. Integrated systems with interoperable data transfer as providable by end-to-end AI solutions can ensure a seamless and swift workflow, giving our patients potentially a quicker and less error-prone path to a diagnosis.

At the very least, even if it is not used for reporting, AI is already used for prostate MRI in the form of deep learning reconstruction, and other systems for planning and performing prostate MRI examinations are around the corner. This adds, and will continue to add, to the standardization of prostate imaging workflow. Another aspect where prostate MRI reporting will be affected in the near future is the use of large language models; there is already evidence that they harbor the potential to structure unstructured reports, cross-check radiological reports for different types of mistakes, and translate reports to other languages or lay/easy language for patients and caregivers [10].

From a stakeholder perspective, AI can be of value for radiologists, reducing workload and potentially improving diagnostic accuracy. Our patients benefit also from more consistent and quicker diagnoses if the MRI pathway can be delivered through AI products [11]. Healthcare administrators will embrace the potentially lower costs and improved efficiency in the diagnostic processes.

## Conclusion

I believe radiologists should be ready for the application of AI in reporting, and prostate MRI already offers the chance to get your feet wet, with applications already on the market. Introducing these solutions now, as an AI pilot program, will help to mitigate the challenges associated with the introduction of new technology, such as how to set up training programs for the applications and how to overcome the potential resistance to change in the workforce.

Ultimately, these systems are cleared from a regulatory standpoint and can, therefore, be used in the daily clinical routine, at least as a preliminary read for later refinement or a confirmatory read in a virtual two-reader setting. Even if these systems are not perfect now, we should be part of the ongoing process of refinement and iterative perfection. The time is now, and we should embrace this opportunity.
